# Paediatric biobanking: Dutch experts reflecting on appropriate legal standards for practice

**DOI:** 10.1007/s00431-016-2810-y

**Published:** 2016-11-19

**Authors:** Elcke J. Kranendonk, Raoul C. Hennekam, M. Corrette Ploem

**Affiliations:** 1 Department of Public Health, Academic Medical Center, University of Amsterdam, Meibergdreef 9, 1105 AZ Amsterdam, The Netherlands; 2 Department of Public Health AMC, Room J2-210, P.O. Box 22660, 1100 DD Amsterdam, The Netherlands; 3 Department of Pediatrics, Academic Medical Center, University of Amsterdam, Meibergdreef 9, 1105 AZ Amsterdam, The Netherlands

**Keywords:** Biobank, Children, Informed consent, Re-contacting, Biological material, Individual findings

## Abstract

Large sets of data and human specimens, such as blood, tumour tissue and DNA, are deposited in biobanks for research purposes, preferably for long periods of time and with broadly defined research aims. Our research focuses on the retention of data and biological materials obtained from children. However important such paediatric biobanks may be, the privacy interests of the children involved and the related risks may not be ignored. The privacy issues arising from paediatric biobanks are the central focus of this article. We first review the international regulations that apply to biobanks and then summarise viewpoints expressed by experts in a round-table discussion. We confine ourselves here to two normative questions: (1) How much control should children’s parents or legal representatives, and later the children themselves, have over the stored materials and data? (2) What should be done if research findings emerge that have serious implications for a child’s health?

*Conclusion*: On the basis of international legal standards and the views of experts, involved in paediatric biobanking, we argue that biological material of children may only be stored in a biobank for scientific purposes if parents provide their explicit consent, the child is re-contacted at 16 or 18 years of age to reconsider storage and use of its material, and the biobank maintains a limited policy in disclosure of individual research findings to the child’s parents.

**Table:** 

**What is Known:**
• *Increasingly*, *biological material of children is stored in biobanks for research purposes.*
• *Clear standards on the conditions under which children*’*s cells or tissues may be stored and used are lacking.*
**What is New:**
• *According to experts*, *storage and use of children*’*s materials should only be allowed if performed in accordance with appropriate consent procedures and feedback policies.*

**Electronic supplementary material:**

The online version of this article (doi:10.1007/s00431-016-2810-y) contains supplementary material, which is available to authorized users.

## Introduction

Collections of health data and human biological materials (such as urine, blood and other tissues) are increasingly required for scientific research in medicine in general. Main reason for this is formed by the progress in technology such as next generation sequencing which allows studying large numbers of samples for biomarkers and other characteristics that are important in basic research and management. The samples are stored for long periods of time in *biobanks* as this will allow using samples repeatedly over time, which is especially important if samples can be obtained only infrequently, typically because the disorder is rare or obtaining samples is infrequently possible. The Royal Dutch Academy of Arts and Sciences defines a biobank as ‘a collection of human biological materials, compiled for scientific or treatment purposes, with linkages to medical, genetic, genealogical or other data about the donors. Biobank data and materials may be, but are not necessarily, stored in the same location. A biobank may therefore also be described as a chain of action consisting of the collection, preparation, storage and use of biological material and personal data’ [23].

There are various types of biobanks. They may differ according to the type of materials they store and the ways they acquire the materials. Two ways of acquisition can be distinguished: samples may be taken from human beings for the specific purpose of scientific research and samples may be obtained via clinical laboratories after analysis for care purposes (‘residual materials’). Another distinction is whether materials are stored in an anonymised, coded, or directly identifiable manner. Few biobanks store completely anonymous materials, as most research requires establishing correlations between samples and other data from participating subjects. If samples and data are stored in an identifiable form (whether coded or non-coded), it is also possible to disclose relevant medical findings to the donors involved.

Although biobanks are vital to health care innovation and improvement, the prolonged storage of personal biological samples entails risks to the individuals involved [2, 10, 13]. Human biological samples may serve a wide range of research purposes, but purposes and their potential results are frequently not known at the time the samples are collected. Samples and data in biobanks contain privacy-sensitive information on the participating donors not only about their current medical condition, but also about their possible health situation in the future. Annas and colleagues described DNA as ‘future diaries’, a source of highly sensitive information in which an important part of each participant’s unique future is inscribed [2]. Data may also have implications for family members. Information in unauthorised hands or used for improper purposes may lead to discrimination and stigmatisation. If sensitive information obtained from samples, such as vulnerability to a serious genetic disorder, disclosure of such information could trigger psychological distress and hamper access to resources such as insurance or employment [19].

Biobanking samples from children is increasing even more rapidly than biobanking of samples of adults, as the percentage of disorders with a genetic background is higher in children compared to that in adults [33]. Indeed several large scale paediatric biobanks are now operating in the Netherlands, such as the Amsterdam-Born Children and Their Development Study (ABCD) [1] and the KOALA cohort study [22].

Next to the general characteristics of biobanks, paediatric biobanks face specific additional problems, such as the young age of donors which prevent responsible decisions at the time of sample collection, and change with age of their decision-making capacity, also legally. The present article explores the issue of how children’s autonomy and rights to privacy ought to be safeguarded when samples are deposited in a research-oriented biobank. Their rights are initially exercised by their legal representatives and later by the children themselves. A considerable lack of clarity still exists on these matters in biobank practice. We shall focus in particular on two questions that have attracted much attention in literature [11, 13, 18, 21, 30]. The first is the right to decide about biobank participation: what rights do a child’s legal representatives have to control whether the child’s samples will be stored in a biobank, and what rights does that child later have upon reaching the legal age of decision-making capacity? The second question concerns how individual research findings are to be dealt with: should these be reported to the child in question (or to a legal representative) in cases findings have implications for the health of the child or of family members? After describing the methods we used in our study (“Used methods” section), we summarise the rules and standards that can be derived from the international legal framework with respect to these two questions (“International legal framework” section). Then the experts’ viewpoints, as expressed during a round-table discussion, are discussed (“Discussing legal standards with experts” section). Taking the latter into account, we suggest a set of principles for paediatric biobanking (“Towards concrete legal standards for banking children’s tissue” section) and close with a few final remarks (“Concluding remarks” section).

## Used methods

We performed a literature search using the electronic databases PubMed, Westlaw International and HeinOnline to select relevant legal documents and literature, using combinations of the following terms: (child OR children OR paediatric OR pediatric), (biobank OR biobanks OR biobanking), (informed consent), (individual findings OR individual results OR incidental findings), (tissue OR biological material), (right to know OR right not to know). The search was restricted to documents in English and Dutch.

Furthermore, we organised a round-table discussion in order to obtain the standards that according to Dutch experts should apply to paediatric biobanks. We aimed to attract participants with their expert knowledge of paediatric biobanking in practice-level or policy-level experience, and participants with a varying background in discipline and profession, including paediatrics, clinical genetics, law and ethics, scientific research and biobank management and policy. Also, a representative of a patient organisation was attracted. We invited 23 experts, of which 11 were unable to be present due to work-related or personal obligations. Twelve experts, working in medical institutes or academic medical centres located in different regions of the Netherlands, were present at the meeting (Table 1) [24]. In preparation of the round-table discussion, we formulated nine normative starting points on the basis of earlier empirical findings, scholarly literature and legal documents related to paediatric biobank research [25]. These starting points were submitted to the experts in advance and were discussed at the meeting one by one to allow experts to express their in-depth views. The organisers of the meeting did not participate themselves in the debate between the invited experts. The discussion was tape-recorded and transcribed verbatim. The key conclusions of the meeting are summarised in paragraph 4. The topics to discuss are available as Supplementary material (Table 2). We would like to stress that the findings of the round-table meeting cannot be seen as fully representative for the whole field of professionals involved in paediatric biobanking. However, the meeting gave us a better picture of what experts involved in research and biobank practice regard as appropriate and well-balanced legal standards in this respect. On the basis of the findings, we were able to reflect on our own, previously formulated, starting points and redefine them as a set of principles for paediatric biobanking.

**Table 1:** Main characteristics of participating experts

	Disciplinairy background experts (*n* = 12)	*n*	Institution
Donor	Patient representative	1	Not applicable
Manager	Biobank manager	1	Academic Medical Center Amsterdam
Physician	Paediatrician	1	University Medical Center Groningen
	Clinical geneticist	4*	Academic Medical Center Amsterdam / Radboud University Medical Center/Maastricht University Medical Center / University Medical Center Utrecht
	Physician-researcher-chair institutional review board	1	Academic Medical Center Amsterdam
Expert	Ethicist	2	University Medical Center Rotterdam/Maastricht University Medical Center
	Lawyer	2	Academic Medical Center Amsterdam/Ministry of Health, Welfare and Sport

*One of the geneticists was also representing the Dutch Society of Clinical Genetics (VKGN)

## International legal framework

First, by far the most important international document is the Convention on Human Rights and Biomedicine (CHRB) from the Council of Europe (1997) [8]. This convention stipulates that storage and use of residual samples may take place only if appropriate information and consent procedures have been fully observed. It is important to note, however, that ‘… sometimes, it will not be possible, or very difficult, to find the persons concerned again in order to ask for their consent. In some cases, it will then be sufficient for a patient or his or her representative, who have been duly informed (for instance, by means of leaflets handed to the persons concerned at the hospital), *not to express their opposition (italics: EJK*, *RCH*, *MCP)*’. In accordance with article 5 and 6 of this convention and the (non-binding) International Declaration on Human Genetic Data, free and informed consent to storage of a child’s residual samples should be authorised by the child’s parents or representatives [8, 32]. Another significant provision of the Biomedicine Convention is that it affirms people’s ‘right to know’ any information collected about their personal health, as well as their ‘right not to know’ (that is, not to be informed of such information if they do not wish to obtain it). Those rights are not absolute and may be overruled in case of vital interest of donors or their relatives (paragraph 70 CHRB Explanatory report) [7].

Second the Organisation for Economic Co-operation and Development (OECD) *Guidelines on Human Biobanks and Genetic Research Databases (HBGRD)* [26] devotes but little attention to the status of children when they reach an age at which they are in principle deemed capable of decision-making or are granted that legal right if their material is still being stored in a biobank. However, paragraph 45 of the CHRB Explanatory Report does note that the opinions of minors should carry increasing weight in decisions in keeping with their age and maturity. A similar principle, affirming children’s right to express their own opinions, is articulated in article 12.1 of the United Nations Convention on the Rights of the Child (UNCRC): ‘States Parties shall assure to the child who is capable of forming his or her own views the right to express those views freely in all matters affecting the child, the views of the child being given due weight in accordance with the age and maturity of the child’ [31]. The OECD guidelines specifically state that consent for continued sample storage and use should be obtained from minors once they gain the capacity to decide according to applicable law or ethical principles (annotation 32) [26].^1^ A comparable principle is proposed by the Public and Professional Policy Committee (PPPC) of the European Society of Human Genetics (ESHG). The PPPC underlined the importance of allowing young donors to renew or withdraw consent [18].

On the basis of the foregoing, the following general framework can be outlined: (1) In principle, residual biological materials and data from children may be deposited for research in biobanks only if their parents or legal representatives have consented to the storage on the basis of adequate information. (2) The information should include details for the parents about how individual research findings are to be dealt with and their opportunity to exercise, on behalf of themselves and the child, the right not to know about such findings. (3) Children at a certain age may also exercise full or partial rights of decision with respect to the retention or continued storage of their materials in a biobank and the disclosure of individual research findings.

## Discussing legal standards with experts

### Informed consent

The first issue addressed was whether the requirement for explicit, general consent from the parents or legal representatives of a young, not yet competent donor is an appropriate means of control over human specimens deposited in paediatric biobanks. Some participants felt that such a requirement was disproportionate, arguing that all patients treated in university hospitals should be aware that scientific research may be performed on their biological materials. Others believed the requirement was defensible or that it depended on the circumstances. ‘*The modalities make a big difference in practice. When you’re talking about consent*, *you have to look at other aspects of the situation too. How does the biobank work? Are the biological materials anonymous or not? How long will they be kept? Can information be disclosed to the donors? Do they have options on whether or not to be informed about certain things? Whether general consent is acceptable also depends on those aspects*’ (legal expert).

Consensus was ultimately reached on the standpoint that long-term storage and use of identifiable residual samples from young donors should in principle be allowed only if the donors’ legal representatives have explicitly consented. In that way, it is best guaranteed that parents can make conscious and responsible decisions about their child’s biobank participation and all the different aspects involved [5, 11, 15, 16]. The experts argued for a concept of ‘generic’ consent, indicating that the parents’ authorization would apply not to a specific research question, but to a particular disease domain. As one expert put it, ‘*If you were to get too specific when you ask for consent*, *you could run into big problems*, *because you*’*d have to re-contact the people every time you want to take a further step in your research. That would be an enormous obstacle*’ (clinical geneticist).

All experts emphasised that clear information to parents is an essential element of an appropriate consent procedure. Specific guidelines were considered necessary, requiring that information be provided in any case on the type of biobank involved, the period of sample retention and the policy on individual findings. Information provided orally could be supported by an information leaflet.

The international legal framework, as described in the “International legal framework” section, leaves room for various consent systems and therefore allows for both explicit and implicit consent systems, and systems that allow exceptions.

### Subsidiarity principle

The need to comply with the subsidiarity principle was also underlined during the round-table discussion, whereby children’s samples are to be retained only if they are essential to the purpose of the biobank in question. As one expert argued: ‘*That principle always applies*, *in fact. Specimens should only be put in a biobank for things that can be studied only with biological materials from that particular category of people*’ (clinical geneticist).

### Child’s right to withdraw

Reservations were aired about the rule enabling children to decide for themselves from age 12 about retention of their samples in biobanks. A joint decision by parents and adolescents seemed more appropriate up to an age of 16 or 18, with independent decisions thereafter. Though a slight preference was apparent for age 18, no clear consensus emerged from the discussion as to the exact age at which donors should decide independently about biobank retention of their samples.

### Disclosure of individual findings

Another main topic treated in the expert meeting was how individual findings should be dealt with, should they emerge during scientific research. Since non-clinical medical research focuses on groups rather than on individuals, typically researchers are not looking actively for individual findings, but they may encounter such findings [27]. The experts agreed that parents and/or children must be informed of any information of a life-saving nature. As one clinical geneticist remarked, ‘*If you run into something in your lab and you know it has individual consequences for the patient involved and that I can avert something terrible by telling them*, *then I don’t think you can expect researchers to keep it to themselves’* (*clinical geneticist*). Participants warned for a misunderstanding of the term ‘duty to warn’, often used in this context, because it suggests a legal duty whereas it merely involves a moral one. In their view biobank, researchers should be aware of the fact that they have a professional moral responsibility to notify the child’s representatives in case of a clear threat to the health of their child.

They pointed to the need to distinguish between contexts in which researchers are at the same time the health care providers of the study participants and those in which they are not. A conservative disclosure policy would suffice in the latter situation. The moral obligation of researchers for early detection and notification applies in particular to findings that are of immediate, unmistakable importance to the donor’s state of health and for which realistic therapeutic or preventative options are indicated. In the literature, views have been expressed that either concur [2, 3, 18] or diverge, with some authors advocating more qualified disclosure policies whereby donors may specify what they do and do not wish to be informed about [4]. According to the experts, a researcher who is also the donor’s health care provider will have to take the legal duty of care for the patient into account as well. Whereas researchers, not being the donor’s health care provider, do not have individual contact with the donor, health care providers do have a treatment relationship. This implies that they have a legal obligation to act according to professional standards, i.e. the duty to take good care of their patient. The latter means, e.g. that findings, resulting from research should be reported to the donor or to the parents even if these do not clearly meet the criteria of immediate health risk and being actionable.

One expert emphasised the importance of clearly communicating in patient information materials which types of findings might be disclosed, so that donors or parents are well aware of what they may and may not expect and have no wrong expectations [14, 20, 29]. Several participants pointed out that the decision of whether or not to disclose of findings to donors or parents is the responsibility of health care providers and not of researchers. The role of the former as care providers enables them to judge best the ways and extends to inform patients.

### Parental right to know and not to know

The next topic was what the parents’ rights to know and not to know should actually entail in relation to research findings made on their children younger than age 12. One expert cautioned that ‘*this is an awkward issue. Shouldn*’*t parents just be allowed to know everything? No*, *wait*, *... parents aren*’*t allowed to stay ignorant of information that*’*s vital to their child*, *so by the same token they shouldn*’*t be able to find out everything*’ (ethicist).

Consensus was ultimately reached: the information provided to parents should not extend the clinically actionable immediate health risks that must be disclosed to donors under the duty to warn. In principle, this means that for instance information about late onset health conditions or about reproductive choices should not be disclosed to parents. The interests of the child ought to be the deciding factor.

The international legal framework does not provide additional guidance: it mentions that the right to know and not to know should be properly implemented in nationals laws and regulations. What role parents (who may have their own interests in knowing or not knowing) should play in that process is left to national discretion. The PPPC emphasised that the potential benefit of children to know actionable findings relevant to their health should prevail over the parental right not to know [18].

### Donors’ rights when reaching the age of decision-making capacity

The expert group discussed the rights of donors who reach the legal age of decision-making capacity while their biological material is still stored in a biobank. Although it is clear from the legal documents that children ought to be given some say as they grow older about whether their samples may be retained and whether they wish to be informed, it cannot be precisely determined at what age those rights may be exercised and to which type of donor control (opt-out or explicit consent) they are entitled. Should biobanks, when donors obtain the competence to decide on these matters (under Dutch law: at the age of 12) simply be required to inform them, or should they actually have to obtain their explicit consent for continued storage and use? The experts judged the latter option as unreasonable. They did not consider adolescents sufficiently capable of deciding about biobank participation until 16 or 18 years of age. A similar view has been expressed in literature [6, 10, 12, 17, 27, 28]. Consequently, they argued that donors should not be asked to consent to continued storage and use until then. They also felt that the consent requirement for adolescents approaching adulthood should not be absolute; exceptions to the requirement should be possible in certain cases, as when very large groups of donors are involved or donors cannot be tracked down or are diseased [12].

It was agreed that a general information letter to donors and their parents is necessary when a child reaches the age of decision-making capacity. One suggestion was ‘*perhaps to send 12-year-olds a card saying*, “*Happy birthday*! *By the time you turn 18*, *we shall*....”’ (physician-researcher-chair institutional review board). Experts proposed that decision-making by the donors could be facilitated by a website offering clear information about the storage and use of their samples, which could be brought under their attention. Notably, the Dutch legal framework permits children aged 12 and older to exercise the right to object to the use of their samples independently, unless they are incompetent to take well-founded decisions. The experts, however, all felt it would go too far to actively inform 12-year-old children about their rights since the matter was seen to be too complex for people of that age to form a good opinion. At the same time, they acknowledged the importance of easily accessible information for young donors and concluded that any special requests from children of that age to terminate their participation should in principle be honoured.

## Towards concrete legal standards for banking children’s tissue

### Parental right to decide about participation

We re-iterate as first principle that biological samples of infants or young children may be deposited in a research-oriented biobank only if the parents have given their explicit informed consent. This principle is articulated in international regulations and is fully endorsed by the expert meeting. The persuasive argument for an explicit-consent procedure is that opt-out procedures do not guarantee that parents are aware of storage and storage circumstances of the residual samples of their children, including major issues such as goals of storage, consequences, potential risks and the voluntary nature of sample donation [24].

We conclude from legal guidelines described in paragraph 3 and the experts’ opinions that children 12 years of age are too young to make a well-founded appraisal of continuation or not of storage of samples in a biobank. Children should be at least 16 to decide independently of its parents about this. The Dutch legislative framework permits adolescents aged 16 and older in principle to decide independently about their treatment and, after acceptance of an amendment bill, about participating in biomedical research.

That children under 16 do not yet qualify for independent consent does not mean, of course, that they do not need to be informed about their participation and should not be involved in the decision-making process. Indeed, they ought to be engaged in the decision by their parents as fully as possible and in keeping with their comprehension abilities. If children then object to biobank participation, their wishes ought to be respected, provided they are deemed capable of making a reasonable decision about the issue. We recall that all experts are Dutch and therefore are familiar with the Dutch legal system which provides fixed age-thresholds for decision-making by the child. Following the experts’ views, three qualifications need to be made with regard to the principle of explicit informed consent. First, in paediatric biobanking, the consent must be generic rather than specific in nature. This implies that the consent does not apply to a specific research question or research protocol. However, consent should be preferably confined to a particular disease domain (i.e. cancer or cardiovascular diseases), because, in that way, the principle of self-determination of young children, who cannot express their own views the moment their material is stored in a biobank, is respected as much as possible. Besides, the donors and their representatives are at least roughly informed about the broader future research goals. Second, understandable information must be provided through an effective website and information leaflet which clearly describes the key characteristics of the biobank, its policies on disclosures of individual research findings and the rights of the child (as exercised by the legal representatives until the child is able to decide independently about participation). Third, exceptions to the consent principle ought to be possible in certain situations; this applies in particular to the requirement to re-contact donors aged 16 whose biological materials were deposited in a biobank at young ages, since such adolescents may now no longer be traceable [24].

### Dealing with individual research findings

Existing international legal documents provide no more than a general framework for acting on research findings that could be vital to individual patients or donors or to their family members. Researchers are to report any important health information they encounter (the right to know), but they are also to respect requests from donors or their parents not to be informed (the right not to know). Neither of these rights is absolute; however; they may be restricted in certain circumstances. With respect to disclosure of individual research findings encountered by researchers, we conclude that, at the minimum, findings that signal a life-threatening situation or serious health detriment for a child, and for which therapeutic or preventative measures are available, are to be reported to the parents and/or the child. This indicates that those findings are not communicated which presage health conditions that are not actionable, especially those that are unlikely to manifest themselves until adult age.

We agree with the experts that a limited disclosure obligation would be the most appropriate, whereby findings would be reported to parents only if they are deemed important and clinically actionable. That would allow no leeway for parents to determine which types of findings they do or do not want to know. A key argument for such a policy is that biobanks do not store samples for the purpose of diagnosing patients (which is the task of a health care provider), but for the purpose of knowledge enhancement. From that standpoint, disclosure of individual findings made in scientific research should be an exception. It is therefore important that parents not have misleading expectations when they consent to have their children’s samples deposited in biobanks [24].

## Concluding remarks

On the basis of the here presented results of the international legal document analysis and the round-table discussion with experts from a wide range of disciplines, we formulated a set of principles that may be useful for professionals, involved in paediatric biobanking. However, we want to stress that there are still several issues in this field we did not address in our research and which need further attention and exploration. To name a few: Does the researcher’s responsibility towards donors include tracing back the donor in case actionable findings emerge, even years after storage of the material? What if, after some time, previously non-actionable findings turn into highly actionable ones? Do researchers have an obligation to screen their data in light of new scientific insights and to look for actionable findings, and if so, in what frequency? Such issues should certainly be part of future research about (paediatric) biobanking.

## Electronic supplementary material

Table 2 (DOC 35 kb)

## Abbreviations

ABCD: Amsterdam-Born Children and Their Development Study
CHRB: Convention on Human Rights and Biomedicine
DNA: Deoxyribonucleic acid
HBGRD: Human Biobanks and Genetic Research Databases
KOALA: Child, parents and health: lifestyle and genetic constitution (in Dutch: Kind, Ouders en gezondheid: Aandacht voor Leefstijl en Aanleg)
OECD: The Organisation for Economic Co-operation and Development
UNCRC: United Nations Convention on the Rights of the Child

## Footnotes

1 See furthermore Article 15 of Council of Europe’s Recommendation R(2006)4 on Research on biological materials of human origin, which stipulates that children must be enabled to exercise their rights as soon as they attain the capacity to provide consent. Included are the rights to withdraw or alter the scope of consent given by parents on their behalf [9].
