# Supplementary material for: Paediatric biobanking: Dutch experts reflecting on appropriate legal standards for practice
Source: Eur J Pediatr. 2016 Nov 19;176(1):75–82. doi: 10.1007/s00431-016-2810-y (PMC5219008; doi:10.1007/s00431-016-2810-y)
Supplement: Supplementary file 1 — (DOC 35 kb) [file 431_2016_2810_MOESM1_ESM.doc]

Supplementary material

| **Table 2** | **Topics to discuss** |
| --- | --- |
| **Informed consent**  **1.** | *The acceptability of long-term storage and use of identifiable residual material of children on the basis of explicit consent of parents or legal representatives .* |
| **2.** | *The generality of consent to allow broad research to a particular disease.* |
| **Information**  **3.** | *The necessity of specific guidelines to providing information to parents.* |
| **Subsidiarity principle**  **4.** | *The subsidiarity principle as a condition under which children’s samples may be retained for biobank purposes.* |
| **Child’s right to withdraw**  **5.** | *Deciding about retention of samples by adolescents together with their parents, and deciding independently by adolescents from 16-18 years old.* |
| **Disclosure of individual findings**  **6.** | *The biobank researcher’s professional responsibility to notify parents or legal representatives in case of individual findings in case of a clear threat to the child’s health.* |
| **7.** | *The sufficiency of a conservative disclosure policy; disclosure of findings with immediate importance to the donor’s health and for which therapeutic or preventive measures are available.* |
| **8.** | *Reference to the policy on disclosure of individual findings in the informed consent process.* |
| **Right to know and not to know**  **9.** | *The scope of parental rights to achieve more or less information on their child than the clinically actionable immediate risks that must be disclosed.* |
| **10.** | *The child’s interests as the deciding factor.* |
| **Donor’s rights when reaching the age of decision-making capacity**  **11.** | *Asking consent to 16- or 18-year-old adolescents to continued storage of their samples and possible exceptions to this requirement.* |
| **12.** | *Informing 12-year-old children through a general information letter about their material being stored in a biobank.* |
